# FKBP4 connects mTORC2 and PI3K to activate the PDK1/Akt-dependent cell proliferation signaling in breast cancer

**DOI:** 10.7150/thno.35561

**Published:** 2019-09-21

**Authors:** Alain Mangé, Etienne Coyaud, Caroline Desmetz, Estelle Laurent, Benoit Béganton, Peter Coopman, Brian Raught, Jérôme Solassol

**Affiliations:** 1 IRCM, INSERM, Univ Montpellier, ICM, Montpellier, France.; 2 Princess Margaret Cancer Centre, University Health Network, Toronto, Canada; 3 BC2M, Univ Montpellier, Montpellier, France; 4 CHU Montpellier, Department of Pathology and onco-biology, Montpellier, France.

**Keywords:** FKBP52, FKBP4, BioID, AKT, breast cancer, mTOR

## Abstract

**Purpose:** Among the FKBP family members, FKBP4 has been described to have a potential role in tumorigenesis, and as a putative tissue marker. We previously showed that FKBP4, an HSP90-associated co-chaperone, can elicit immune response as a tumor-specific antigen, and are overexpressed in breast cancer.

**Experimental design:** In this study, we examined how loss of FKBP4 affect breast cancer progression and exploited protein interactomics to gain mechanistic insight into this process.

**Results:** We found that FKBP4 expression is associated with breast cancer progression and prognosis, especially of ER-negative breast cancer. Furthermore, FKBP4 depletion specifically reduces cell growth and proliferation of triple negative breast cancer cell model and xenograft tumor model. Using specific protein interactome strategy by BirA proximity-dependent biotin identification, we demonstrated that FKBP4 is a novel PI3K-Akt-mTOR proximal interacting protein.

**Conclusion:** Our results suggest that FKBP4 interacts with PI3K and can enhance Akt activation through PDK1 and mTORC2.

## Introduction

Following stimulation through hormones, growth factors or nutrients, the PI3K/PDK1/Akt signaling pathway becomes activated and regulates cell proliferation, cell cycle, and survival 1. Upon ligand binding, specific receptors recruit Akt and PDK1 to the plasma membrane 2. The subsequent PDK1- and mTORC2-driven phosphorylation of Akt Thr308 3 and Ser473 residues 4, respectively, are both required to activate Akt.

Akt has been shown to contribute to oncogenesis through triggering growth and cell proliferation via multiple downstream pathways. mTOR, a critical regulator of Akt, forms two different complexes: mTOR complex 1 (mTORC1) and mTORC2. mTORC1 regulates cell growth and proliferation by phosphorylating downstream translational effectors (e.g. p70 ribosomal S6 kinase 1 (S6K1) and eukaryotic initiation factor 4E (eIF4E) binding protein 1 (4E-BP1)) to coordinately upregulate mRNA translation, ribosome biogenesis, protein synthesis and cell growth 5, 6. mTORC2 regulates cell survival and proliferation by Akt Ser473 phosphorylation 4. Small molecules, such as growth factors and hormones, have been described to activate Akt, mTORC2, and subsequently mTORC1 through an Akt-dependent phosphorylation. Nutrients, such as amino acids, have also been described to activate Akt and mTORC2 7, and stimulate mTORC1 directly via an Akt-independent phosphorylation mechanism 8, 9. Under nutrient-rich conditions, activated mTORC1 inhibits autophagy through direct phosphorylation of Ulk1 10. Another critical Akt substrate is the glycogen synthase kinase-3 beta (GSK3β), which phosphorylation triggers its inactivation 11. GSK3β drives cell proliferation by regulating stability and synthesis of proteins involved in the G1/S cell cycle phase transition (e.g. cyclin D1 12), hence playing a central role in a number of transforming mechanisms when deregulated.

There is a growing body of evidence indicating that molecular chaperones regulate key signaling molecules 13, including nuclear hormone receptors, protein tyrosine kinases, serine /threonine kinases such as Akt 14, 15, cell cycle and cell death regulators, that control the stability, activation, and subcellular localization of major signal transducers. Immunophilins constitute a family of molecular chaperones and intracellular receptors targeted by immunosuppressive drugs such as cyclosporine A, FK506/tacrolimus or rapamycin/serolimus. This family encompasses FK506-binding proteins (FKBPs) and cyclophilins 16. In addition to their well-established roles in T-cell activation, when complexed with their ligands, FKBPs are involved in numerous cellular functions, such as protein folding and stability, cellular signaling and protein trafficking 16. For example, some members of the FKBP family are co-chaperones of the heat shock protein 90 (HSP90) complex. This complex facilitates the folding, activity and proteolytic stability of a broad range of client proteins (see http://www.picard.ch/Hsp90Int/index.php). In a previous study, our group showed that FKBP4 (alias FKBP52), an HSP90-associated co-chaperone, is a tumor-specific antigen capable of eliciting an immune response in breast cancer 17. Moreover, we and others have reported that FKBP4 was found up-regulated in breast cancer tissues and breast cancer cell lines, both at the mRNA and protein levels 17-19. This upregulation was particularly noticeable in estrogen receptor-positive tissues or cells 18, 19. As a co-chaperone, FKBP4 regulates the activity of several client proteins including steroid hormone receptors (*e.g.* androgen (AR), glucocorticoid (GR), progesterone (PR), mineralocorticoid, and estrogen receptors (ER)) 20, nuclear factors (NF-kB) 21, and others proteins such as Argonaute 2 (AGO2) 22 or Tau 23.

Here we demonstrate for the first time that FKBP4 is associated with breast cancer progression and prognosis, especially of ER-negative breast cancer. We observed that inhibition of FKBP4 expression alters cell growth in a triple negative (ER-, PR-, and HER2-negative) breast cancer cell model and a murine xenograft tumor model. To gain mechanistic insight on the role of FKBP4 in this cancer subtype, we used mass spectrometry to characterize the *in vivo* proximal interactome of FKBP4 and demonstrated that FKBP4 is a novel PI3K/PDK1/mTORC2 proximal interacting protein. Our results suggest that FKBP4 promotes Akt phosphorylation through PI3k/PDK1 and mTORC2, and that is plays an important role in cell growth and proliferation. These findings could be important for the triple negative breast cancer patients who do not respond to hormonal therapy and have few treatment options.

## Materials and Methods

### Patients

For immunohistochemistry (IHC) experiments, 28 normal breast, 20 carcinomas *in situ* (CIS) and 67 invasive breast cancer (IBC) tissues were obtained from the Pathology department of Institut Universitaire du Cancer de Toulouse. All patients signed an informed consent permitting analyses of tissues and a local ethic committee approved the analysis of the human breast cancer tissues. Detailed clinical and pathological information on the associated breast cancer patients are listed in Table 1.

### Immunohistochemistry

RCL2-fixed, paraffin-embedded tissue sections were used for the immunohistochemistry (IHC) analyses for human breast cancer tissue. Both deparaffinization and epitope retrieval were performed with the PT Link system (Dako), following manufacturer's instructions. Briefly, sections were treated for 45 min at 95°C with Tris/EDTA *p*H9 antigen retrieval buffer. Endogenous peroxidase activity was quenched before the blocking step of non-specific binding sites performed with the EnVisionTM FLEX blocking reagent (Dako). Sections were then incubated with FKBP4 polyclonal antibody (ProteinTech,1:200) for 20 min at RT. Following washes, detection of antibody binding was visualized with a peroxidase-conjugated polymer backbone using diaminobenzidine as a chromogen. Sections were counterstained with hematoxylin. Scores were obtained by estimating average signal intensity (0, none; 1+, mild; 2+, moderate; 3+, intense) and the percentage of positive cells (0-100%). The intensity and percentage scores were then combined to give the overall IHC score (low, medium, high).

### Cell culture

MDA-MB-231 cells were grown in 10% fetal calf serum (FCS) DMEM/F12/Glutamax (Gibco). *In vitro* transfection of small interfering (si)RNA was performed using Dharmafect 2 reagent (Dharmacon). siGENOME SMARTpool, constituted of 4 siRNAs directed against human FKBP4, and non-targeting control siRNA were purchased from Dharmacon. Briefly, cells were plated to reach 30-40% confluency, and were transfected once or twice (at a 24 h interval), according the manufacturer's instructions. Experiments were performed from 72 h post-transfection. For starvation conditions, cells were grown for 16 h in DMEM/F12, and incubated with 10% FCS for 1 h, except for specific stimulations where cells were incubated for 1 h without amino acids in PBS, before stimulation with DMEM/PBS supplemented with insulin (100 nM), EGF (100 ng/mL), or doubled amino acid concentration (mix of essential amino acids and GlutaMAX^TM^ from Gibco). For shRNA-mediated gene suppression, lentiviral particles containing shRNA targeting human FKBP4, or non-targeting sequence, were synthesized by the Montpellier Vectorology platform. Viral supernatants were added to MDA-MB-231 cells for 24 h, before puromycin selection. FKBP4 knockdown was verified by Western blot. For mass spectrometry (MS) analysis, proximity-dependent biotin identification (BioID) 24 was carried out essentially as we described previously 25. In brief, the full-length human FKBP4 (BC001786) coding sequence was amplified by PCR and cloned into our pcDNA5 FRT/TO FLAGBirA* expression vector. Using the Flp-In system (Invitrogen), HEK293 T-REx Flp-In cells stably expressing FLAGBirA* alone or FLAGBirA*-FKBP4 were generated. After selection (DMEM + 10% FCS + 200 µg/mL Hygromycin B), 10 x 150 cm2 plates of sub-confluent (60%) cells were incubated for 24 h in complete medium supplemented with 1 µg/mL tetracycline (Sigma-Aldrich), 50 µM biotin (BioShop). Cells were collected (2,000 rpm, 3 min). The pellets were washed twice with PBS, dried and snap frozen.

### Growth curves

Cells were transfected twice with FKBP4- or non-targeting siRNAs, then seeded at 20,000 cells/well in 24-well plates in quaduplicate, the day of the second transfection (stated as T0 in Figure 2). Cells were then trypsinized and counted everyday using an automated cell counter (Z1^TM^ Coulter Counter).

### Cell cycle analysis

Cells were transfected twice with FKBP4 or non-targeting siRNAs, then seeded in 6-well plates 72 h post-infection. The day after, cells were incubated with 30 µM 5-bromo-2-deoxyuridine (BrdU) for 30 min. Cells were then harvested, washed in PBS, and fixed in ice-cold 75% ethanol. Fixed cells were then digested with pepsin (0.05% in 30 mM HCl) for 20 min at 37°C, and washed twice in PBS. Finally, cells were incubated with the anti-BrdU antibody (Oxford Biotechnology) for 1 h at 37°C, followed by IgG-FITC conjugated antibody (Souther Biotech) for 30 min in the dark. After washing, cells were counterstained with propidium iodide (25 µg/ml). Samples were analyzed in triplicates using a FC-500 flow cytometer (Coulter).

### Apoptosis

Apoptosis was detected using the Annexin V/7-AAD kit (Coulter). Briefly, transfected cells were plated in 6-well plates, 72 h post-transfection. Then, cells were washed with cold PBS, and resuspended in ice-cold 1X binding buffer, followed by addition of 10 µl Annexin V-FITC and 20 µl 7-AAD. After 15 min at room temperature in the dark, 400 µl 1X binding buffer were added, and cells were analyzed by flow cytometry using a FC-500 flow cytometer (Coulter) in triplicates.

### Western-blot

Total proteins were extracted using 1X cell lysing buffer (Cell Signaling Technology). Samples were separated by electrophoresis in SDS- polyacrylamide gels (12%) and transferred to PVDF membranes. Membranes were blocked for 1 h with PBST (PBS plus 0.1% Tween-20) containing 5% non-fat milk. Blots were incubated overnight at 4°C with primary antibodies in PBST containing 5% BSA at the manufacturer's recommended dilution. The antibodies were purchased from the following suppliers: FKBP4 (ProteinTech); anti-pS473 Akt, anti-pT308 Akt, anti-Akt, anti-pT286 Cyclin D1, anti-Cyclin D1, anti-E2F-1, anti-pT37/46 4E-BP1, anti-pS9 GSK-3β, anti-GSK-3β, and anti-mTOR (Cell Signaling Technology); anti-GAPDH and anti-Actin (Santa Cruz Biotechnology); anti-Tubulin and anti-PIK3R2 (Sigma-Aldrich). After washing, blots were incubated with either an anti-rabbit HRP, or an anti-mouse HRP-conjugated antibody (Cell Signaling Technology) for 1 h at 25°C. After washing, blots were developed with Supersignal WEST Pico Plus (Life Technologies SAS).

### *In vivo* tumorigenesis assay

All procedures were conducted in the IRCM animal facility and in accordance with the French guidelines for experimental animal studies (C34-172-27). All efforts were made to minimize suffering. Six-week-old athymic nude mice were obtained from Harlan (n=11 per group). A total of 2 x 10^6^ MDA-MB-231 transducted cells were resuspended in 150 µl DMEM/F12 and injected subcutaneously. Tumor growth was monitored twice the week. Animals were sacrificed 50 days post-graft by CO2 asphyxiation, and tumor were removed for further analysis.

### Biotin-streptavidin and FLAG affinity purification for MS

For BioID, the cell pellet was resuspended in 10 mL of lysis buffer (50 mM Tris-HCl pH 7.5, 150 mM NaCl, 1 mM EDTA, 1 mM EGTA, 1% Triton X-100, 0.1% SDS, 1:500 protease inhibitor cocktail (Sigma-Aldrich), 1:1000 benzonase nuclease (Novagen)) and incubated on a rotator at 4°C for 1 h, briefly sonicated to disrupt visible aggregates, and centrifuged at 45,000 x g for 30 min at 4°C. Supernatant was transferred to a new 15 mL conical tube. 30 µL of packed, pre-equilibrated streptavidin sepharose beads (GE) were added and the mixture incubated for 3 h at 4°C under rotation. Beads were pelleted by centrifugation at 2,000 rpm for 2 min and transferred with 1mL of lysis buffer to a fresh Eppendorf tube. Beads were washed once with 1 mL lysis buffer and twice with 1 mL of 50 mM ammonium bicarbonate (pH=8.3). They were then transferred to ammonium bicarbonate in a new centrifuge tube and washed two times with 1 mL ammonium bicarbonate buffer.

The FLAG tag-based immunoprecipitation (FLAG tag-based IP) was performed as previously described. Briefly, the cell pellet was resuspended in 1:4 pellet weight/lysis buffer (50 mM HEPES-NaOH pH 8.0, 100 mM KCl, 2 mM EDTA, 0.1% Nonidet P-40, 10% glycerol, 1 mM PMSF, 1 mM DTT and 1:500 protease inhibitor cocktail (Sigma-Aldrich) and incubated on ice for 10 min, subjected to one freeze-thaw cycle, then centrifuged at 27000 g for 20 min at 4 °C. Supernatant was transferred to a fresh 15 ml conical tube, 30 µl packed, pre-equilibrated FLAG-M2 agarose beads (Sigma-Aldrich) were added and the mixture was incubated for 2 h at 4 °C with end-over-end rotation. Beads were pelleted by centrifugation at 2,000 rpm for 2 min and transferred with 1mL of lysis buffer to a fresh Eppendorf tube. Beads were washed once with 1 mL lysis buffer and twice with 1 mL of 50 mM ammonium bicarbonate (pH=8.3). Elution was performed by incubating the beads twice with 150 µl of 125 mM ammonium hydroxide (pH 11). Eluate was transferred to a fresh Eppendorf tube and lyophilized before trypsin digestion.

Tryptic digestion was performed by incubating the beads with 1 µg MS-grade TPCK trypsin (Promega, Madison, WI) dissolved in 200 µL of 50 mM ammonium bicarbonate (pH 8.3) overnight at 37°C. The following morning, 0.5 µg MS-grade TPCK trypsin was added, and beads were incubated 2 additional hours at 37°C. Beads were pelleted by centrifugation at 2,000 x g for 2 min, and the supernatant was transferred to a new Eppendorf tube. Beads were washed twice with 150 µL of 50 mM ammonium bicarbonate, and these washes were pooled with the first eluate. The sample was lyophilized and resuspended in buffer A (0.1% formic acid). 1/5th of the sample was analyzed per MS run.

### Mass spectrometry

Analytical columns (75-µm inner diameter) and pre-columns (150-µm inner diameter) were made in-house from fused silica capillary tubing from InnovaQuartz (Phoenix, AZ) and packed with 100 Å C18-coated silica particles (Magic, Michrom Bioresources, Auburn, CA). Peptides obtained after biotin-streptavidin affinity purification (see extended Supplementary Materials and Methods) were subjected to liquid chromatography (LC)-electrospray ionization-tandem mass spectrometry, using a 120 min reversed-phase (100% water-100% acetonitrile, 0.1% formic acid) buffer gradient running at 250 nL/min on a Proxeon EASY-nLC pump in-line with a hybrid LTQ-Orbitrap Velos mass spectrometer (Thermo Fisher Scientific, Waltham, MA). A parent ion scan was performed in the Orbitrap using a resolving power of 60,000, then up to the twenty most intense peaks were selected for MS/MS (minimum ion count of 1000 for activation), using standard collision induced dissociation fragmentation. Fragment ions were detected in the LTQ. Dynamic exclusion was activated such that MS/MS of the same m/z (within a range of 15 ppm; exclusion list size = 500) detected twice within 15 s were excluded from analysis for 30 s. For protein identification, Thermo RAW files were converted to the mzXML format using Proteowizard 26, then searched using X!Tandem 27 against the human (Human RefSeq Version 45) database. X!Tandem search parameters were: 15 ppm parent mass error; 0.4 Da fragment mass error; complete modifications, none; cysteine modifications, none; potential modifications, +16@M and W, +32@M and W, +42@N-terminus, +1@N and Q. Each of the two biological replicates of FlagBirA*-FKBP4 samples was analyzed using two technical replicates. Data were analyzed using the trans-proteomic pipeline (TPP) 28 via the ProHits software suite 29. Proteins identified with a Protein Prophet cut-off of 0.9 were analyzed with the SAINT express algorithm (v3.3) 30. Fourteen control runs (consisting of Flag-BirA*only) were collapsed to the four highest spectral counts for each prey, and the SAINT score cut-off value was set to 0.80.

### Statistical analysis and gene ontology analysis

Differences between conditions were analyzed using two-tailed Student's t test or Mann-Whitney U test. A p-value of <0.05 was chosen as statistical significance threshold. Kaplan-Meier survival curves for the relationship between relapse-free survival (RFS) and FKBP4 mRNA expression in breast cancer was performed using the online tool (http://kmplot.com/) 31. Gene ontology (GO) classification, including KEGG pathway, GO cellular function and GO cellular component categories, was determined using the DAVID bioinformatics resources (https://david-d.ncifcrf.gov/) 32. The significance of the gene-term enrichment was determined using a modified Fischer's exact test (EASE score), which ranks the overrepresented GO processes. The STRING (http://string-db.org/) database (version 10.0) was utilized to search reported protein-protein interaction network within the FKBP4 interactome 33.

## Results

### FKBP4 expression in normal and cancerous breast tissues

**Figure 1:**
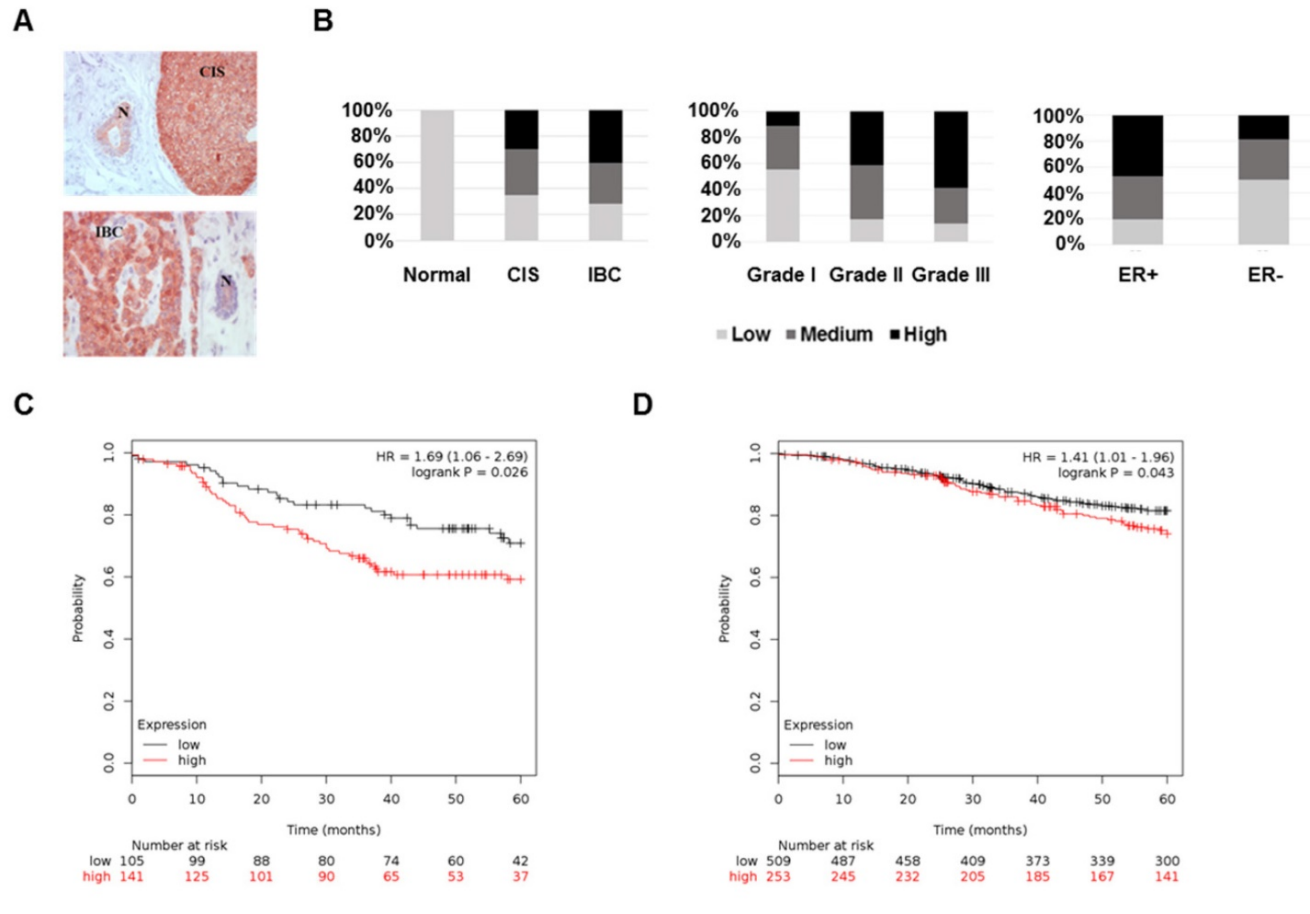
The high expression of FKBP4 in breast cancer is associated with cancer progression and prognosis. **(A)** Representative immunohistochemistry of FKBP4 in human breast cancer tissue. Normal ducts (N), carcinoma *in situ* (CIS) and invasive breast cancer (IBC) are indicated. Original magnification, x 200. **(B)** Staining distribution of FKBP4 for normal tissues and breast tumors, histologic grade, and ER status are shown and are based on overall IHC score (low, medium, high). The overall IHC score is a combination of the intensity and percentage of positive cells scores. **(C)** Relapse-free survival of breast cancer patients with ER/PR-negative breast tumors relapsing or not before 5 years. Patients with high FKBP4 mRNA expression (red) exhibited a shorter RFS interval (*P=*0.026). **(D)** Relapse-free survival of breast cancer patients with ER-positive breast tumors relapsing or not before 5 years. Patients with high FKBP4 mRNA expression (red) exhibited a shorter RFS interval (*P=*0.043).

To test FKBP4 differential expression across breast tissues, we performed IHC on breast tumors and CIS samples. FKBP4 IHC analysis of 115 patients revealed a FKBP4 immunostaining localized predominantly in the cytoplasm and nucleus of normal, *in situ*, and invasive breast cells (Figure 1A). FKBP4 expression levels were strongly and significantly increased in cancerous breast cells compared to surrounding normal breast cells (*P*<0.001, Figure 1B). The rate of moderate to high FKBP4 expression was significantly higher in CIS (65.0%, 13/20) or IBC (71.6%, 48/67) than in surrounding normal breast tissues (0%, 0/28). We then assessed the correlation between FKBP4 expression and clinical features. The rate of moderate to high FKBP4 expression was significantly higher in grade 2 (82.8%, 29/35) and grade 3 (85.7%, 30/35) than in grade 1 (46.7%, 7/15) breast tumors (*P*<0.01, Figure 1B). FKBP4 expression was higher in the estrogen receptor (ER)-positive (80.3%, 53/66) than in ER-negative breast tissues (52.4%, 11/21) (*P*<0.05, Figure 1B). However, no correlation was observed between FKBP4 expression and other clinical characteristics (Table 1), such as age, stage, lymph node status, progesterone receptor or HER2 overexpression.

To assess the predictive significance of FKBP4 expression on 5-years relapse-free survival (RFS), we extracted the FKBP4 mRNA expression levels (low vs. high) related to RFS from publicly available microarray data set. In the ER/PR-negative breast tumor data set (n=246), patients with high FKBP4 expression exhibited a shorter and significant RFS interval (Log-rank, *P*=0.026) (Figure 1C). Similar result was obtained with the ER-positive breast tumor data set (n=762; Log-rank, *P*=0.043) (Figure 1D).

Based on these results, we hypothesize that high expression of FKBP4 is associated with breast cancer progression, and more particularly, that FKBP4 might be a biomarker of poor prognosis in patient with ER/PR-negative breast tumor.

### Modulation of FKBP4 expression alters cell growth and cell cycle in breast cancer cells

**Figure 2:**
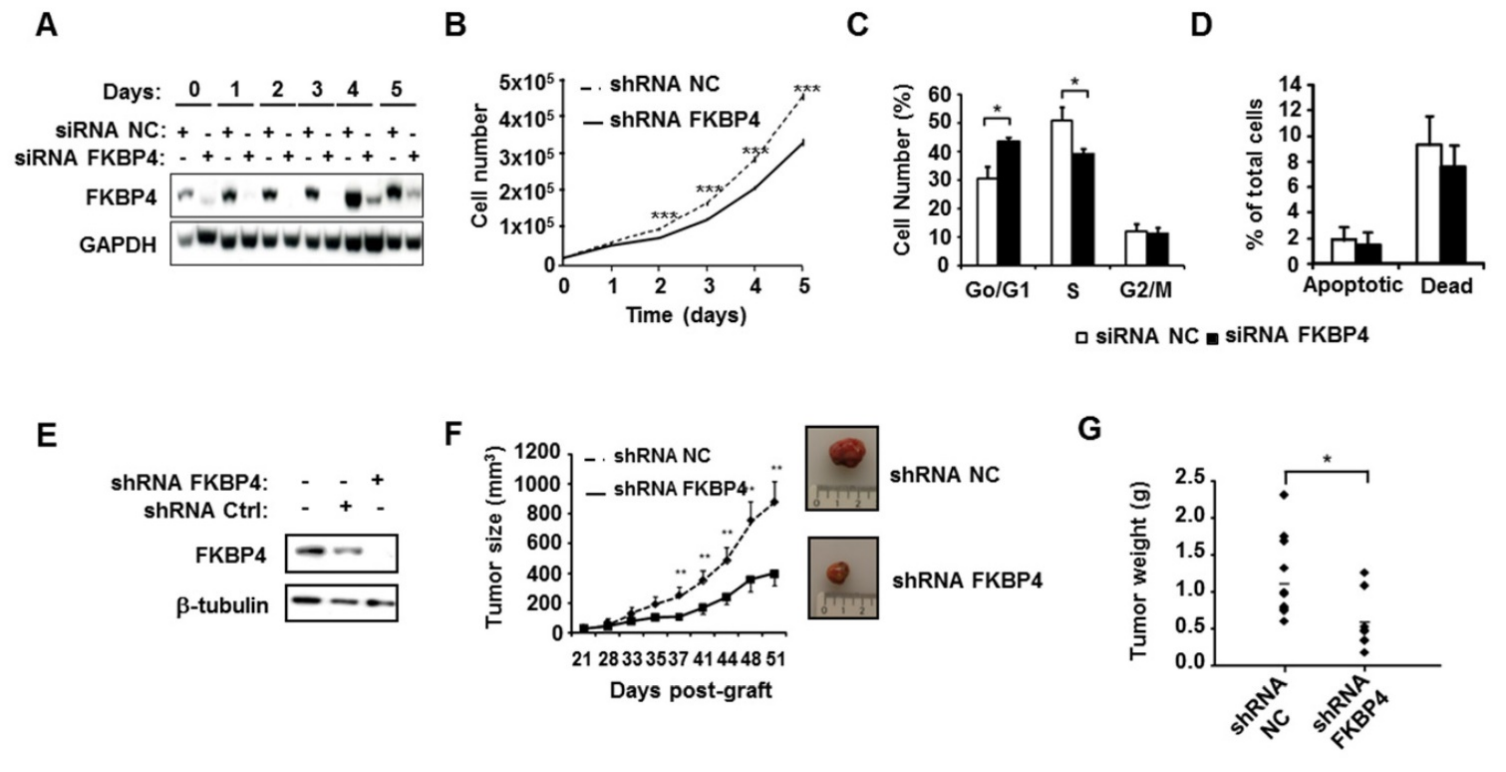
FKBP4 knockdown impairs cell growth and proliferation *in vitro* and *in vivo*. **(A)** To verify knock-down efficiency, FKBP4 protein expression in MDA-MB-231 cells was analyzed by immunoblotting. Cells were transfected with FKBP4 or negative control (NC) siRNA, plated 3 days after transfection and analyzed dayly during 6 days. **(B)** The cell proliferation rate was determined by cell count every day during 6 days. **(C)** Cell cycle was analyzed using flow cytometry in MDA-MB-231 cells transfected with FKBP4 or NC siRNA. **(D)** Apoptosis rate was determined using flow cytometry and Annexin-V/7-AAD staining in MDA-MB-231 cells transfected with FKBP4 or NC siRNA. Apoptotic cells are defined by Annexin-V positive and 7-AAD negative cells. Dead cells are defined by Annexin-V negative and 7-AAD positive cells. **(E)** MDA-MD-231 cells were stably transduced with FKBP4 or negative control shRNA. FKBP4 protein expression was analyzed by immunoblotting at the time of injection. **(F)** MDA-MB-231 cells expressing FKBP4 or NC shRNAs were grafted subcutaneously into athymic nude mice (n=11 per group). Tumor volume was monitored twice a week for 50 days. Representative tumors of each groups were photographed at day 50. **(G)** At day 50, mice were sacrificed and tumor weight was determined. For cell proliferation, cell cycle and apoptosis assays, data are based on 3 independent experiments. Results are shown as mean ± SEM. * *P*<0.05; ** *P*<0.01; *** *P*<0.001.

To assess the potential role of FKBP4 in breast cancer progression, the impact of silencing FKBP4 was evaluated on the triple negative MDA-MB-231 cell line. As shown in Figure 2A, FKBP4 expression was inhibited over 160 h by the FKBP4 siRNA, as compared to the negative control siRNA. The effect of FKBP4 depletion on MDA-MB-231 cell growth were then monitored over a similar time period. Cell growth kinetics were measured by counting the number of transfected cells during 5 days. We observed a decrease in cell growth in FKBP4 siRNA transfected cells compared to negative control siRNA transfected cells (Figure 2B). This decrease was significant from day 2 (12% inhibition, *P*<0.001) to day 5 (30% inhibition, *P*<0.001). These results clearly indicate that the expression level of FKBP4 impacts the proliferation of MDA-MB-231 cancer cells. Same results were obtained with 3 other human breast cancer cell lines, of which two are ER/PR-positive (MCF-7 and T47D), and one is triple negative (MDA-MB-436) (Supplementary Figure S1).

The consequences of FKBP4 inhibition on the cell cycle progression were analyzed in transfected MDA-MB-231 cells by flow cytometry (Figure 2C). FKBP4 siRNA transfected cells showed a significant increase of cells in G0/G1 phase compared to negative control siRNA transfected cells (43.5% and 30.7% respectively; *P*<0.001). This increase in the G0/G1 phase is correlated with a significant decrease in S phase (39.2% and 50.8%, respectively; *P*=0.0013), with no difference in G2/M phase (*P*=0.6012). Finally, to estimate the contribution of apoptosis to the cell count drop observed following FKBP4 knockdown, we performed annexin V/7-AAD staining followed by flow cytometry analysis (Figure 2D). We observed no significant difference in FKBP4 siRNA transfected cells compared to negative control siRNA transfected cells on percentage of apoptotic cells (annexin V positive) or necrotic cells (annexin V negative/7-AAD positive). Together, these results demonstrate that the knockdown of FKBP4 can inhibit cell growth *in vitro* by blocking the cell cycle in G0/G1.

### FKBP4 knockdown inhibits tumor cell growth *in vivo*

To test the involvement of FKBP4 in tumor growth *in vivo*, we established a MDA-MB-231 cell line-derived xenograft (CDX) tumor model in athymic mice, by injecting cells stably transfected with FKBP4-targeting shRNA, or with non-targeting shRNA. FKBP4 shRNA expressing cells showed a complete loss of protein at Western-blot level compared to the control cells (Figure 2E). These cells were then injected subcutaneously in nude mice, and the tumor growth monitored over a month, starting at day 21 (detection of a palpable tumour; Figure 2F). The subcutaneous tumor size reached 884 mm^3^ 51 days post-graft in the control group, while the FKBP4 shRNA CDX tumor grew much slower, reaching 399 mm^3^ at day 51 (*P<*0.01). These results were correlated with the tumor weight at day 51 (Figure 2G), which was significantly decreased in FKBP4 shRNA group compared to control group (0.587 g and 1.160 g, respectively; *P*=0.021). These results demonstrate that the depletion of FKBP4 inhibits MDA-MB-231 CDX growth *in vivo*.

### Identification of FKBP4 interactors

To explore the mechanism of FKBP4 in breast cancer, we identified the FKBP4 proximal proteins using the recent BioID technique (Supplementary Figure S2). Briefly, FKBP4 was fused with an abortive mutant of the E.Coli BirA biotin ligase (BirA R118G; termed BirA*) 24. This fusion bait protein recapitulates the interactions of endogenous FKBP4, and biotinylates free epsilon amine groups of protein partners within a radius of 10 nm. Tetracyclin-inducible HEK293T-REx Flp-In stable cell lines expressing FlagBirA*-FKBP4 or FlagBirA* alone were incubated 24 h in presence of biotin and tetracyclin. Cell lysates were then subjected to streptavidin affinity purification to trap the biotinylated proteins. After removal of the background using the SAINT algorithm, we identified 114 high-confidence proximal interactors of FKBP4 (Supplementary Table S1). Amongst these, 5 were previously reported as FKBP4 interactors in BioGRID and IntAct database 34, 35, including the glucocorticoid receptor ARHGAP35, glomulin (GLMN; a FKBP-associated protein), NUDCD3 (that maintains the stability of the dynein intermediate chain), Plastin 1 (PLS1; an actin filament binding protein) and PIK3C2A (the catalytic subunit of PI3K), validating our methodology.

**Figure 3:**
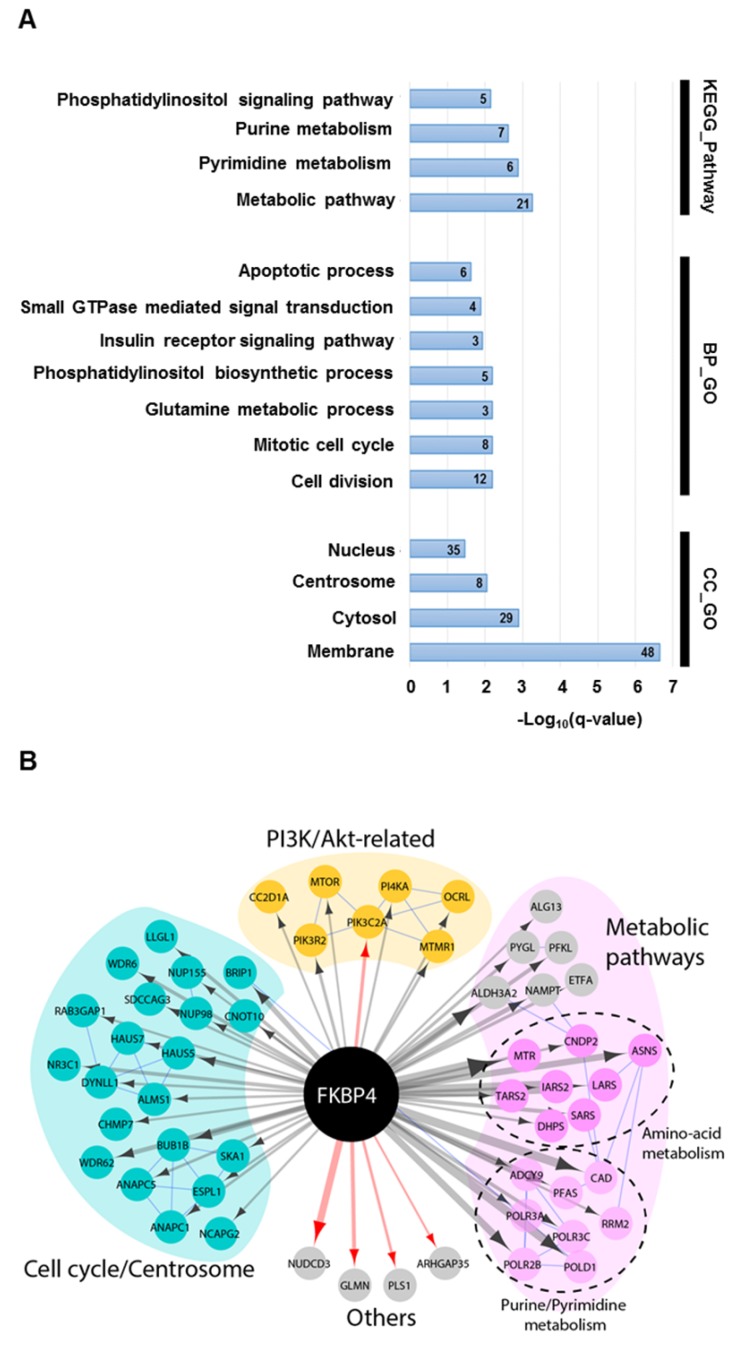
The FKBP4 interactome was analyzed using BioID.** (A)** Significant enrichment of Gene Ontology (GO) terms based on KEGG pathway, biological process (BP) and cellular compartment (CC) were shown. Number of proteins per GO term are indicated. **(B)** Protein-protein interaction network of FKBP4 interactome was analyzed using STRING database. Functional enrichment in the network confirms significant enrichment in metabolic pathways, in cell division and mitotic cell cycle pathway and PI3K/Akt-related pathway, which links FKBP4 with critical regulators of these GO terms.

Forty per cent of the identified interactors were primarily annotated as cytosolic, 29% nuclear, 24% membrane-associated, and 7% centrosomal (Figure 3A). GO analysis on biological process and KEGG pathway showed a significant enrichment in metabolic pathways (*e.g.* purine, pyrimidine or glutamine metabolisms), in cell signaling pathways (*e.g.* Phosphatidylinositol signaling system), and in cell cycle (Figure 3A). The interactome map presented in Figure 3B integrates the main results of this analysis and provide an overview of the interactions between the detected proximal prey proteins. Interestingly, the phosphatidylinositol signaling pathway group of interactors links FKBP4 with critical regulators of cell growth and proliferation through the PI3K/PDK1/Akt signaling pathway. This protein group comprises mTOR, the essential catalytic core of two distinct complexes: mTORC1 and mTORC2 8; the phosphatidylinositol 3-kinase PI3K with its regulatory and catalytic subunits (PI3KR2 and PIK3C2A) 36, which generates the phospholipid second messenger molecule PIP3 allowing the recruitment of PDK1 and Akt to the membrane 2; CC2D1A, a scaffold protein interacting with Akt, PDK1 and EGFR 37; and MTMR1, a member of the myotubularin-related protein family with 3-phosphoinositide phosphatase activity, which regulates Akt signaling 38. In attempt to confirm some of these interactors, we did a standard FLAG tag-based immunoprecipitation (Flag IP-MS). After removal of the background, we identified 64 high confidence interactors of FKBP4 (Supplementary Table S2), from those only 4 were identified with the BioID approach. Among them, PIK3C2A was previously identified as a partner of FKBP4 using the same IP-MS approach 39. Together, the BioID and FLAG IP-MS data suggested that PI3K could be the anchor point of FKBP4, and could explain, with the other proximal interactors, the anti-oncogenic properties of FKBP4 silencing identified above.

### FKBP4 affects phosphorylation and activation of Akt

**Figure 4:**
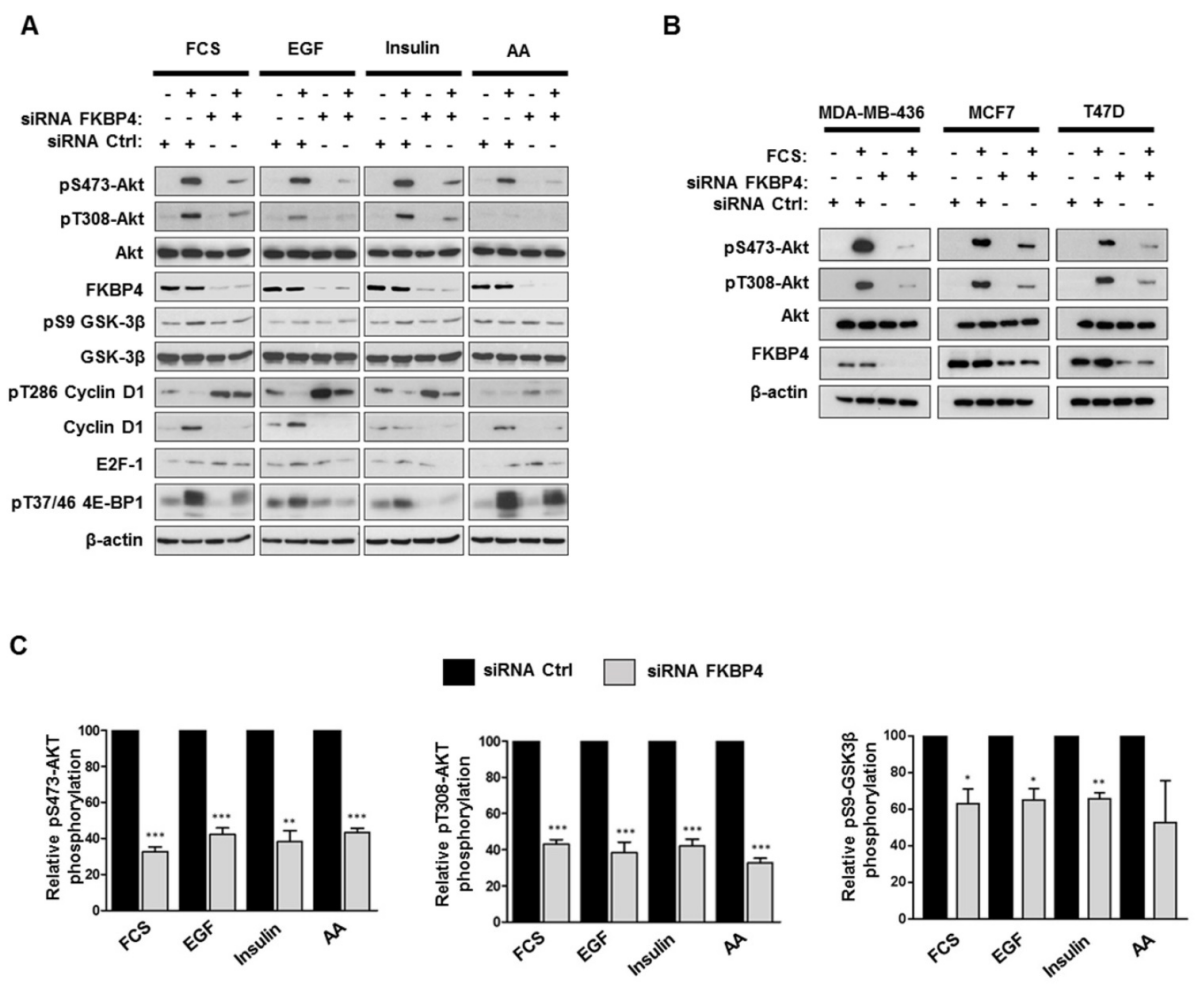
FKBP4 affects phosphorylation of Akt at Ser473 and Thr308, and activation of the PI3K/Akt signaling pathway. **(A)** MDA-MB-231 cells, transfected with FKBP4 or negative control siRNA, were serum deprived overnight and stimulated with 10% FCS for 1 h, or after serum deprivation, cells were deprived of amino acids with PBS for 2 h, followed by stimulation with 100ng/mL EGF, 100 nM insulin or 2X amino acids for 1 h. Cell lysates were analyzed by immunoblotting using the antibodies indicated. **(B)** MDA-MB-436, MCF7 and T47D cells were treated as in A. The phosphorylation of Akt in cell lysates were detected by Western blot. **(C)** Quantification of Western blot presented in A was performed for pS473-Akt, pT308-Akt and pS9-GSK-3β. * *P*<0.05; ** *P*<0.01; *** *P*<0.001.

To analyze the role of FKBP4 on PI3K/PDK1/Akt signaling pathway, MDA-MB-231 cells were transfected with FKBP4-targeting or negative control siRNA, incubated overnight in DMEM/F12 without FCS, followed by a 1 h stimulation with FCS. Cell lysates were then analyzed by immunoblot to detect Akt phosphorylation at Ser473 and Thr308 (Figure 4A). In the control cells, we observed an increase of phospho-Ser473 and phospho-Thr308 Akt in the presence of FCS. In FKBP4 silencing conditions, we observed significantly decreased levels of both phospho-Ser473 and phospho-Thr308 Akt (by 60-80% as compared with the FCS-stimulated control cells; *P* < 0.001). Same results on Akt phosphorylation were obtained with the 3 other human breast cancer cell lines (MCF-7, T47D, and MDA-MB-436) (Figure 4B), regardless of the single FKBP4 siRNAs used (Supplementary Figure S3). Given that a range of hormones, growth factors and nutrients (such as amino acids) have been described to regulate the PI3K/Akt signaling and activate Akt, mTORC1 and mTORC2 1, we evaluated the consequences of FKBP4 knockdown on Akt phosphorylation after stimulation by different factors such as EGF, insulin and amino acids. As previously mentioned, siRNA transfected MDA-MB-231 cells were incubated overnight in DMEM/F12 without FCS, followed by a 1 h incubation in PBS (for amino acid deprivation), and then stimulated with EGF (100 ng/mL), insulin (100 nM) or amino acids (2X) for 1 h. Under these conditions, FKBP4 silencing decreased both phospho-Ser473 and phospho-Thr308 Akt levels after insulin or EGF induction (Figure 4A). Upon amino acid stimulation of cells transfected with control siRNA, we observed a clear increase of phospho-Ser473 Akt levels and a slight increase of the phospho-Thr308 Akt signal intensity. Similarly to EGF and insulin induced cells, we demonstrated that FKBP4 is required for amino acid-induced Akt phosphorylation at Ser473 and Thr308. These results indicate that FKBP4 participates in Akt activation in response to multiple type of stimuli (growth factors or nutrients). Moreover, these results demonstrate that FKBP4 promotes PI3K/PDK1- and mTORC2-mediated Akt phosphorylation (corresponding to the phospho-Thr308 and phospho-Ser473 levels, respectively).

Activation of Akt is known to stimulate multiple proliferation pathways. Among its downstream targets, GSK3β and mTORC1 could potentially drive cell proliferation through the regulation of stability and synthesis of several proteins involved in cell cycle 12, 40, 41. We therefore analyzed the phosphorylation levels of cyclin D1 and 4E-BP1 (respectively targeted by GSK3β and mTORC1) in FKBP4 knockdown MDA-MB-231 cells. As shown in Figure 4A, the phosphorylation levels of GSK3β were increased in siRNA control-treated cells after stimulation with FCS, EGF and insulin, whereas FKBP4 knockdown resulted in a 20-30% decreased phosphorylation as compared to the stimulated control cells. The phosphorylation of cyclin D1, which serves as a primary signal for its proteasomal degradation 12, was strongly increased in FKBP4 knockdown cells, and the total amount of cyclin D1 was strongly reduced as compared to the stimulated control cells. We next examined the mTORC1 activation status by evaluating the phosphorylation levels of its downstream signaling target, 4E-BP1, that plays a predominant role in cell proliferation 42. FKBP4 knockdown caused a marked decrease in the phosphorylation of 4E-BP1 after stimulation with FCS, EGF and insulin, as compared to the stimulated control cells (Figure 4A). In contrast, we did not observe a decreased phosphorylation of 4E-BP1 after amino acid stimulation of cells, suggesting an Akt- and FKBP-independent phosphorylation of 4E-BP1 through the Rag-ragulator-mediated translocation of mTORC1 to lysosomal membrane 8. These observations suggest that the effect of FKBP4 on the regulation of the PI3K/Akt pathway is partially mediated by mTORC2, but not by mTORC1.

## Discussion

Among the FKBP family members, FKBP4 has been described to play a potential role in tumorigenesis, and as a putative marker. FKBP4 is expressed in most tissues, with a minimal level in breast, bladder and testis 20, 43. Interestingly, elevated levels of FKBP4 were observed in several cell line models of hormone-dependent cancer, including breast cancer cell lines 18, 19 and prostate cancer cell lines 44. In previous studies, we and others have observed a higher expression of FKBP4 in breast cancer tissues and pre-invasive breast cancer as compared to normal breast tissues 17, 18. Similar observations were reported in prostate biopsy tissues 45 and hepatocellular carcinoma 46, where FKBP4 was proposed to be a tumor biomarker. In the present study, we characterized the expression levels, the clinical correlations, and new role of FKBP4 in pathogenesis of breast cancers.

To evaluate the differential expression of FKBP4 in breast tissues, we used IHC to compare its expression in breast cancer and surrounding normal breast tissues. As previously reported in breast cancer cell lines, we confirmed that FKBP4 expression was significantly higher in ER-positive tissues than in ER-negative tissues. To our knowledge, higher levels of FKBP4 in ER-negative breast cancer tissues compared to surrounding normal breast cells have not previously been described. Interestingly, FKBP4 expression was also strongly associated with higher histologic grade. A moderate-to-high FKBP4 expression was detected in the majority of grade 2 (82.8%) or grade 3 (85.7%) tumors, compared to grade 1 (46.7%), demonstrating that FKBP4 is associated with faster growing, moderately or poorly differentiated grade breast cancer, and an aggressive phenotype. Moreover, by analyzing the mRNA expression levels of FKBP4, we revealed a significant association between its expression in ER/PR-negative, as well as in ER-positive breast tumors, and the RFS, demonstrating the prognostic potential of FKBP4 in predicting the patients' outcome. The lower correlation between FKBP4 mRNA expression and RFS in ER-positive breast tumors could be explain by other major covariables such as ER expression itself which is the main indicator of endocrine therapy response. However, these findings remain to be validated at the protein level, in a new cohort and correlated with a complete clinical characterization. Altogether, these results suggest that FKBP4 may be a useful biological marker to estimate breast cancer progression and prognosis, and may functionally play a role in promoting breast cancer growth in ER-positive and more interestingly in ER/PR-negative tumors.

FKBP4 was originally found to be associated with other co-chaperones, such as FKBP5 (alias FKBP51), CyP40 and the protein phosphatase PP5, within steroid receptor-HSP90 heterocomplexes 20. The FKBPs-HSP90 complex regulates the steroid receptor signaling and is potentially involved in a variety of steroid-based diseases. In the context of hormone-dependent cancers, the overexpression of FKBP4 could strongly impact the development of the disease through the AR and ER functions. In prostate cancer cell lines, FKBP4 was described as positive regulators of AR-mediated cell growth 20, 47. The physiological impact of FKBP4 on ER activity in FKBP4 knockout mice 48, 49, as well as its role in breast cancer, were not clearly demonstrated, despite: (i) its strong correlation with ER expression 17, 18; (ii) the upregulation of its gene expression by estrogen 19; and (iii), its association with ER/HSP90 complexes 20. In this study, we inhibited FKBP4 in the highly aggressive, poorly differentiated and triple negative breast cancer cell line MDA-MB-231. Our data showed that inhibition of FKBP4 altered cancer cell growth *in vitro* by inducing a G1 phase arrest. We confirmed this effect on cell proliferation in a CDX tumor model *in vivo*, where the down-regulation of FKBP4 significantly impaired tumor growth. Hence, these data suggested that FKBP4 has a strong impact on growth activity in breast cancer through an ER-independent mechanism, at least in these ER-negative models. To elucidate the molecular mechanism underlying the FKBP4 function, we explored the molecular neighborhood of FKBP4 using the BioID approach. As an alternative for co-immunoprecipitation, BioID reveals the proximal protein-protein interaction network in live cells, and has been successfully used to map transient and dynamic interactions of many proteins (reviewed in 50). We identified 114 high confidence proximal interactors of FKBP4, including known interactors, and providing a rich set of new candidate partners. FKBP4 proximal interactome was significantly enriched in interactors involved in metabolic pathways, cell division, and cell signaling pathways, comprising critical regulators of cell growth and proliferation. This latter group suggest a link between FKBP4 and the PI3K/PDK1/Akt signaling pathway through the proximal interactors PI3K, CC2D1A and mTOR. Interestingly, using standard IP-approach, we confirmed the interaction of FKBP4 with the catalytic subunit of PI3K, PIK3C2A, suggesting that the interaction between FKBP4 and PI3K could be central in the functional impact of FKBP4 in breast cancer cells. Akt signaling pathway plays a key role in cellular homeostasis and is often dysregulated in human cancers, resulting in the perturbation of the cell cycle, cell growth and survival. We hypothesize that FKBP4 could promote Akt activation through PI3K interaction and the other proximal interactors identified by BioID. Our data support this hypothesis, showing that FKBP4 acts as a positive regulator of Akt-mediated cell growth in different ER-positive and ER-negative cell lines. After stimulation by different factors such as FCS, EGF or insulin, we found that FKBP4 was required for the phosphorylation of key regulatory residues within Akt: in the activating loop of the catalytic domain at Thr308, and in the COOH-terminal domain at Ser473. Upon amino acid stimulation, FKBP4 modulated principally the mTORC2-mediated Akt phosphorylation at Ser473. Phosphorylation of both sites leads to fully activated Akt, that stimulates multiple downstream effectors. Our results suggest that inhibition of FKBP4 induces a cell cycle arrest in triple-negative breast cancer cells through down-regulation of the PI3K/Akt signaling activity, resulting in Cyclin D1 downregulation. Cyclin D1, a key molecule in cell cycle progression and cell proliferation, is required for G1-to-S phase transition and its inhibition induces cell cycle arrest in the G1 phase (as observed upon FKBP4 knockdown). Cyclin D1 regulation is partially linked to mitogenic stimulation via the PI3K/Akt signaling pathway. At the protein level, it has been reported that GSK3β mediates cyclin D1 phosphorylation at Thr286, which serves as a primary signal involved in its proteasomal degradation 12. At the mRNA level, the phosphorylation of 4E-BP1 causes the release of eIF4E, thus allowing cap-dependent translation of many target mRNAs including cyclin D1 51. Our results indicate that FKBP4 inhibition leads to cyclin D1 degradation *via* the decreased GSK3β and 4E-BP1 phosphorylation, and the increased GSK3β-mediated phosphorylation of cyclin D1.

Mechanistically, many studies have reported that FKBP4 and FKBP5 exhibit an antagonistic role in the functions and activities of the steroid receptors GR, PR or mineralocorticoid receptors 52, 53, 54 . FKBP5 is preferentially associated with inactive GR and PR, and has an inhibitory effect on the steroid hormone receptor functions. Interaction between hormone and its receptor induces FKBP5 to FKBP4 substitution, resulting in the nuclear translocation of receptor complexes and transcriptional activation of target genes. The transcriptional activity of NF-κB, a nuclear factor subject to nucleocytoplasmic shuttling, is also controlled by a switch between FKBP4 and FKBP5, but independently of the HSP90 chaperone 21, 55. More interestingly, it has been also reported that the antagonist protein FKBP5 is a negative regulator of cell growth by inhibiting the PI3K/Akt signaling pathway, which can influence the response to chemotherapy 14. FKBP5 acts as a scaffold protein and forms complexes with Akt and PHLPP, the phosphatase that specifically dephosphorylates Akt at Ser473 14. Taken into account this former study, our findings suggest that a antagonist role of FKBP4 and FKBP5 could be a critical step in the regulation of PI3K/Akt signaling pathway. In this hypothesis, PI3K is activated by growth factor signaling. PI3K converts phosphatidylinositol-4,5-bisphosphate (PIP2) into phosphatidylinositol-3,4,5-triphosphate (PIP3), a critical second messenger that recruits Akt and PDK1 to the plasma membrane 2, as well as, mTORC2 56. CC2D1A forms a complex with Akt, PDK1 and EGFR to promote the Thr308 PDK1-mediated phosphorylation of Akt 37. Akt is also phosphorylated at the Ser473 residue by mTORC2 4. We therefore hypothesize that FKBP4 facilitates PIP3 formation through its interaction with PI3K and facilitate the recruitment of the other proximal interactors (CC2D1A/PDK1/mTORC2/Akt) resulting in fully activated Akt. Finally, an interchange of FKBP4 by FKBP5 could induce a modification in the complexes, resulting in the recruitment of PHLPP and the inactivation of Akt.

The BioID approach allowed us to refine the molecular environment of FKBP4 and oriented our research towards its possible impact on the Akt/mTOR pathway. This BioID dataset includes a mixture of direct and indirect interactors, as well as, very proximal proteins. Several biological parameters could impact the identification of interactors as the distance between the bait and the prey or the number of accessible residues for the biotinylation. All of these render the validation step very challenging. Standard IP-MS approaches could be used in this way but with a lot of important limitations 57, 58. However, there are many examples, as in our study, where IP-MS fails to detect an interaction while BioID can 25, 57. We estimate that the generally fast kinetics of low stoichiometry of phosphorylation events limits the BioID approach to catch all the interactors of a phosphorylation complex upon its activation. Attractive new and faster tools (TurboID) have been recently developed and will allow for an additional level of characterization in a near future 59.

In summary, our data suggest that FKBP4 significantly potentiates the Akt signaling in triple-negative breast cancer. Dysregulation of the Akt signaling pathway affects cell cycle and apoptosis and is unambiguously linked to cancer development and response to therapy. Overexpression of FKBP4 could thus contribute to tumorigenesis by increasing proliferation and tumor growth. These findings will have important consequences on cancer etiology, and more particularly in triple-negative breast cancer where FKBP4 could be a promising therapeutic target.

## Figures and Tables

**Table 1:** Clinicopathologic characteristics of breast carcinomas

	CIS	IBC
Characteristics	*N*=20 (%)	*N*=67 (%)
**Age (years) median, [min-max]**	66 [38-86]	73, [37-97]
**Histotype**		
Ductal	19 (95.0)	55 (82.1)
Lobular	1 (5.0)	12 (17.9)
**Tumor size**		
T1	11 (55.0)	32 (47.7)
T2	8 (40.0)	31 (46.3)
T3	1 (5.0)	1 (1.5)
T4	1 (5.0)	3 (4.5)
**Histological grade**		
I	3 (15.0)	12 (17.9)
II	12 (60.0)	23 (34.3)
III	3 (15.0)	32 (47.8)
Missing	2 (10.0)	0
**Lymph node status**		
Negative	10 (50.0)	34 (50.7)
Possitive	9 (45.0)	30 (44.8)
Missing	1 (5.0)	3 (4.5)
**Estrogen Receptor**		
Negative	1 (5.0)	20 (29.9)
Positive	19 (95.0)	47 (70.1)
**Progesterone Receptor**		
Negative	2 (10.0)	22 (32.8)
Positive	9 (45.0)	37 (55.2)
Missing	9 (45.0)	8 (12.0)
**Her-2 overexpression**		
Negative	14 (70.0)	48 (71.6)
Positive	1 (5.0)	7 (10.5)
Missing	5 (25.0)	12 (17.9)
		
