# Supplementary material for: FKBP4 connects mTORC2 and PI3K to activate the PDK1/Akt-dependent cell proliferation signaling in breast cancer
Source: Theranostics. 2019 Sep 21;9(23):7003–15. doi: 10.7150/thno.35561 (PMC6815969; doi:10.7150/thno.35561)
Supplement: Supplementary file 1 — Supplementary figures and tables. [file thnov09p7003s1.pdf]

Supplementary Information for

FKBP4 connects mTORC2 and PI3K to activate the PDK1/Akt-dependent cell proliferation signaling in breast cancer

Alain Mangé, Etienne Coyaud, Caroline Desmetz, Benoit Béganton, Peter Coopman, Brian Raught, Jérôme Solassol

Corresponding author: Alain Mangé, IRCM – INSERM U1194, 208 rue des Apothicaires, 34298 MONTPELLIER CEDEX 5.

Tel: (33)467612412, Fax: (33)467339590, alain.mange@umontpellier.fr

This PDF file includes:

- Figs. S1, S2 and S3
- Tables S1, S2

Figure and Table legends

Fig. S1. FKBP4 knockdown impairs cell growth and proliferation in two ER/PR-positive cell lines, MCF-7 (**A**) and T47D (**B**), and one triple-negative cell line, MDA-MB-436 (**C**). The cell proliferation rate was determined by cell count every day during 6 days. Data are based on 3 independent experiments. Results are shown as mean ± SEM. * P<0.05; ** P<0.01; *** P<0.001.

Fig. S2. BioID workflow chart.

Fig. S3. Effect of single FKBP4 siRNAs on the phosphorylation of Akt at Ser473. MDA-MB-231 cells, transfected with negative control or 4 single FKBP4 siRNAs (constitutive of the SMARTpool used in the study), were serum deprived overnight and stimulated with 10% FCS for 1 h. Cell lysates were analyzed by immunoblotting. The phosphorylation of Akt was examined using the antibody indicated.

Table S1. FKBP4 BioID-based interactome. The proteins identified are from two biological replicates and two technical replicates. Total spectral count (sum of the spectral identified from the four MS runs), and the corresponding SAINT score is specified.

Table S2. FKBP4 FLAG tag-based IP-MS interactome. The proteins identified are from two biological replicates and two technical replicates. Total spectral count

(sum of the spectral identified from the four MS runs), and the corresponding SAINT score is specified.

Fig. S1.

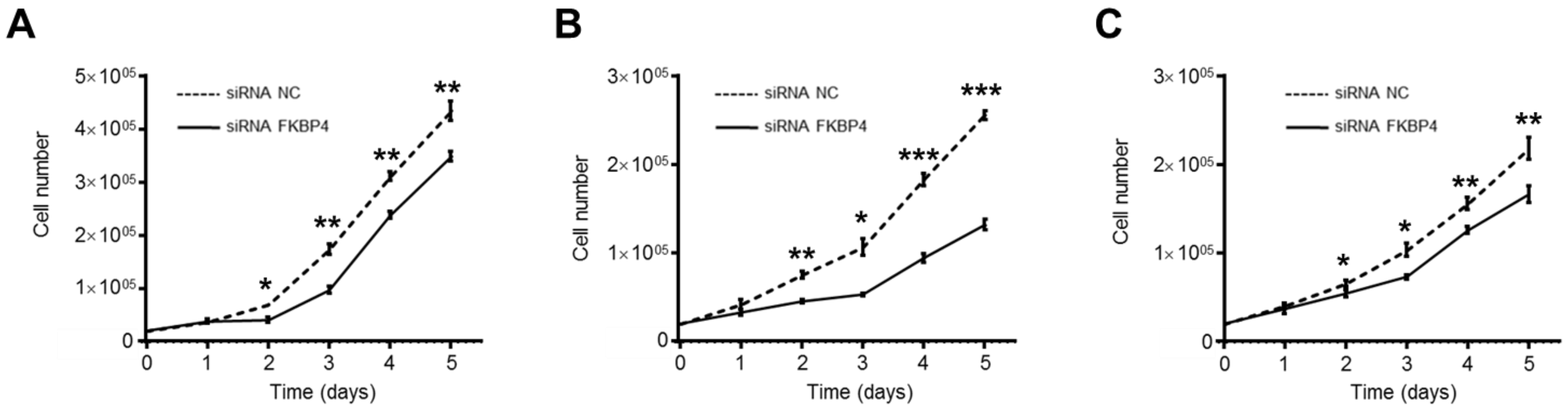
A
B
C
siRNA NC
siRNA FKBP4
Cell number
Time (days)
5×10^05
4×10^05
3×10^05
2×10^05
1×10^05
0
0 1 2 3 4 5
*
**
***

Fig. S2.

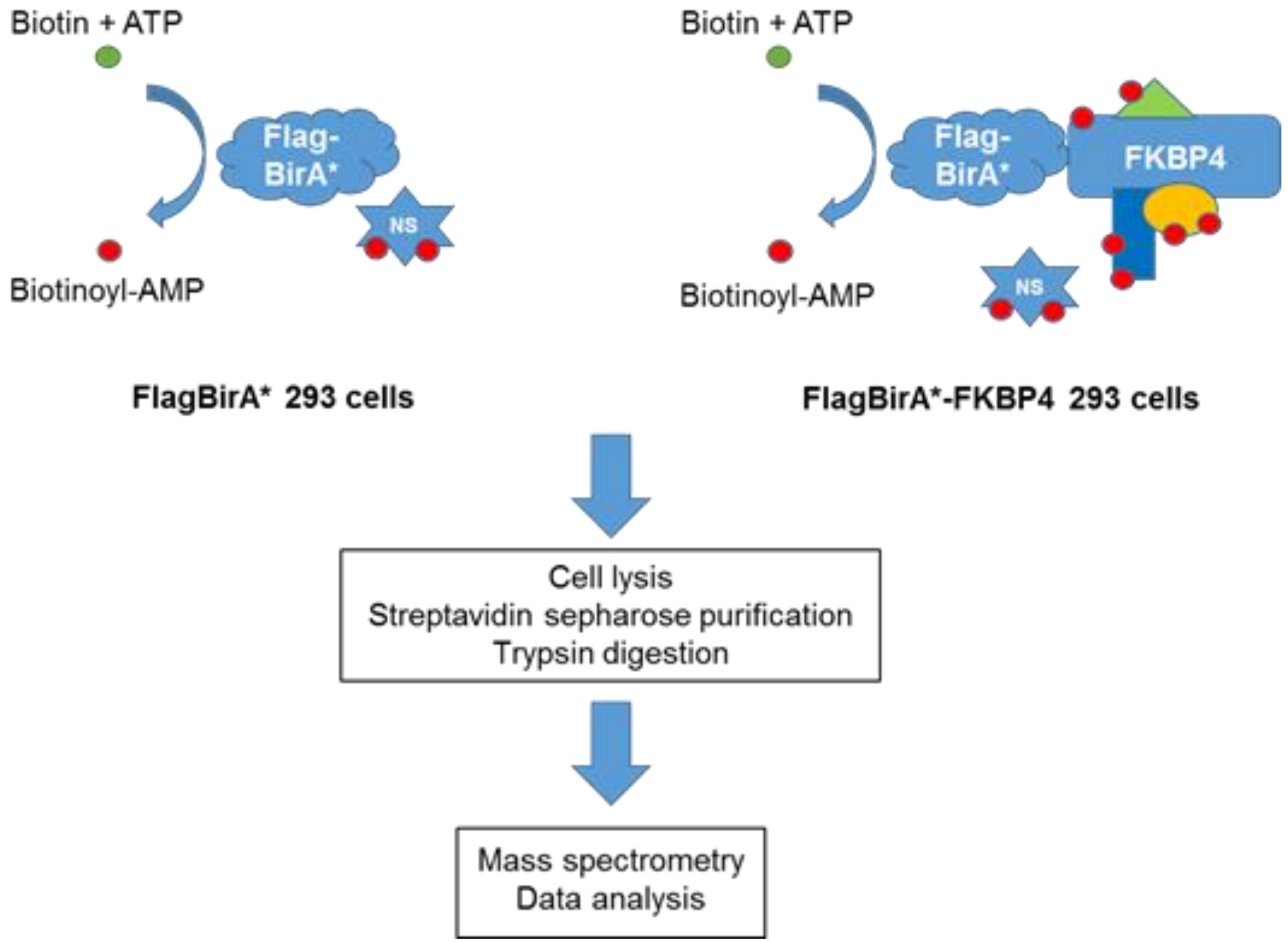
Biotin + ATP
Flag-
BirA*
NS
Biotinoyl-AMP
FlagBirA* 293 cells
Biotin + ATP
Flag-
BirA*
FKBP4
NS
Biotinoyl-AMP
FlagBirA*-FKBP4 293 cells
Cell lysis
Streptavidin sepharose purification
Trypsin digestion
Mass spectrometry
Data analysis

Fig. S3.

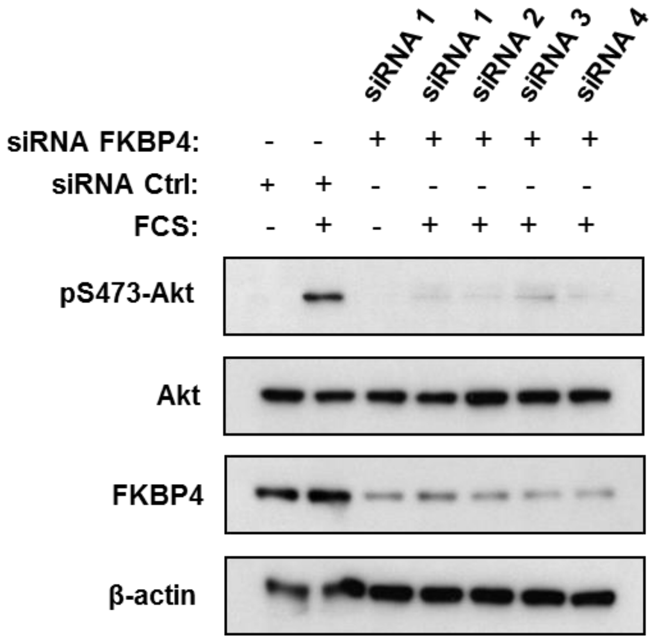

siRNA 1
siRNA 1
siRNA 2
siRNA 3
siRNA 4
siRNA FKBP4: - - + + + + +
siRNA Ctrl: + + - - - - -
FCS: - + - + + + +
pS473-Akt
Akt
FKBP4
β-actin

Table S1.

			BioID		
Uniprot ID	Protein Name	Gene name	Total controls	Total FKBP4	SAINT
Q9NY65	TBA8	TUBA8	0	234	1.00
Q99707	METH	MTR	3	195	1.00
Q9P2J5	SYLC	LARS	44	174	1.00
Q9H6T3	RPAP3	RPAP3	38	158	1.00
P51648	AL3A2	ALDH3A2	28	118	1.00
P27708	PYR1	CAD	12	109	1.00
P28340	DPOD1	POLD1	4	107	1.00
Q9BQ52	RNZ2	ELAC2	3	85	1.00
Q8IVD9	NUDC3	NUDCD3	0	85	1.00
P30876	RPB2	POLR2B	0	83	1.00
Q8N3C0	ASCC3	ASCC3	0	80	1.00
O15067	PUR4	PFAS	4	76	1.00
P08243	ASNS	ASNS	13	73	1.00
Q9H3U1	UN45A	UNC45A	5	72	1.00
P61221	ABCE1	ABCE1	16	68	0.99
O60566	BUB1B	BUB1B	13	57	0.96
Q9BX63	FANCJ	BRIP1	8	51	1.00
Q12979	ABR	ABR	1	48	1.00
Q9ULE6	PALD	KIAA1274	11	46	0.96
P43490	NAMPT	NAMPT	13	44	0.93
O43379	WDR62	WDR62	2	44	1.00
O94927	HAUS5	HAUS5	11	43	0.91
Q6NSI4	CX057	CXorf57	11	39	0.81
Q9C0B7	TNG6	TMCO7	0	37	1.00
O94763	RMP	URI1	0	37	1.00
P49591	SYSC	SARS	0	36	1.00
P17858	PFKAL	PFKL	4	33	0.95
Q92990	GLMN	GLMN	0	33	1.00
Q9P2D0	IBTK	IBTK	3	33	0.95
O75153	CLU	KIAA0664	3	32	0.95
P04150	GCR	NR3C1	8	32	0.81
Q9BUI4	RPC3	POLR3C	3	28	0.99
Q9H9A6	LRC40	LRRC40	1	27	1.00
Q9NNW5	WDR6	WDR6	0	27	0.90
Q96RR4	KKCC2	CAMKK2	0	26	1.00
Q15334	L2GL1	LLGL1	0	26	1.00
Q6AI08	HEAT6	HEATR6	0	25	1.00
O00443	P3C2A	PIK3C2A	0	25	1.00
Q96N67	DOCK7	DOCK7	0	24	1.00
Q9Y2I1	NISCH	NISCH	0	23	1.00
Q7L576	CYFP1	CYFIP1	0	22	1.00
O95219	SNX4	SNX4	2	22	0.95
P13804	ETFA	ETFA	0	22	0.86

Q9H9T3	ELP3	ELP3	0	21	1.00
P06737	PYGL	PYGL	0	21	1.00
Q9Y4W6	AFG32	AFG3L2	0	20	1.00
O00459	P85B	PIK3R2	0	20	1.00
Q96MX6	WDR92	WDR92	0	20	1.00
Q15477	SKIV2	SKIV2L	0	18	1.00
Q5VV42	CDKAL	CDKAL1	0	18	1.00
Q9BVM2	DPCD	DPCD	0	18	1.00
Q9Y312	AAR2	C20orf4	1	18	0.99
Q9UK61	F208A	FAM208A	2	18	0.90
Q5VZ89	DEN4C	DENND4C	0	17	1.00
Q9Y2Z2	MTO1	MTO1	0	17	1.00
Q8WVJ2	NUDC2	NUDCD2	0	17	1.00
Q9H9A5	CNO10	CNOT10	1	17	0.99
Q9NSE4	SYIM	IARS2	1	17	0.97
Q9Y6Y8	S23IP	SEC23IP	2	17	0.86
Q13613	MTMR1	MTMR1	0	16	1.00
Q9BWH6	RPAP1	RPAP1	0	16	1.00
Q9UJX4	APC5	ANAPC5	1	16	0.99
Q14651	PLSI	PLS1	0	16	0.98
Q9UBB6	NCDN	NCDN	0	15	1.00
Q5THK1	PR14L	PRR14L	0	15	1.00
Q15436	SC23A	SEC23A	0	15	1.00
Q9NVH1	DJC11	DNAJC11	2	15	0.85
Q9Y4W2	LAS1L	LAS1L	0	14	0.99
Q9UBK9	UXT	UXT	0	14	0.99
Q9UBB4	ATX10	ATXN10	0	14	0.98
Q9H1A4	APC1	ANAPC1	2	14	0.81
O14802	RPC1	POLR3A	0	13	1.00
Q8TEU7	RPGF6	RAPGEF6	0	13	0.99
Q6YN16	HSDL2	HSDL2	1	13	0.96
Q96KP4	CNDP2	CNDP2	0	13	0.88
Q8IZP0	ABI1	ABI1	2	13	0.85
Q8TCU4	ALMS1	ALMS1	0	12	1.00
Q68DQ2	CRBG3	CRYBG3	0	12	1.00
P63167	DYL1	DYNLL1	0	12	1.00
Q9P2P1	NYNRI	NYNRIN	0	12	0.99
Q96C92	SDCG3	SDCCAG3	0	12	0.99
Q96BD8	SKA1	SKA1	0	12	0.99
A0A024R8K8	A0A024R8K8	HELZ	0	12	0.96
Q7Z4G4	TRM11	TRMT11	0	11	0.99
Q14674	ESPL1	ESPL1	0	11	0.97
Q9BW92	SYTM	TARS2	0	11	0.97
Q96IX5	USMG5	USMG5	0	11	0.97
Q01968	OCRL	OCRL	0	11	0.95
P49366	DHYS	DHPS	0	10	0.97
Q8N1I0	DOCK4	DOCK4	0	10	0.97
O60503	ADCY9	ADCY9	0	9	0.97
J3KN10	J3KN10	PI4KA	0	9	0.97
P51784	UBP11	USP11	0	9	0.97

P52948	NUP98	NUP98	1	9	0.86
O95639	CPSF4	CPSF4	0	8	0.95
Q86XI2	CNDG2	NCAPG2	0	8	0.95
Q9NP73	ALG13	ALG13	0	8	0.93
Q8WUX9	CHMP7	CHMP7	0	8	0.93
Q9NYP7	ELOV5	ELOVL5	0	8	0.93
Q15042	RB3GP	RAB3GAP1	0	8	0.93
P49458	SRP09	SRP9	0	8	0.93
Q9BZV1	UBXN6	UBXN6	0	8	0.93
P31350	RIR2	RRM2	0	8	0.86
O75694	NU155	NUP155	0	7	0.95
Q9NRW7	VPS45	VPS45	0	7	0.95
Q6P1N0	C2D1A	CC2D1A	0	7	0.86
Q8NB46	ANR52	ANKRD52	0	6	0.93
P36543	VATE1	ATP6V1E1	0	6	0.93
Q14191	WRN	WRN	0	6	0.86
P43304	GPDM	GPD2	0	6	0.84
Q9NRY4	RHG35	ARHGAP35	0	5	0.84
Q99871	HAUS7	HAUS7	0	5	0.84
Q8NDC0	MISSL	MAPK1IP1L	0	5	0.84
P42345	MTOR	MTOR	0	5	0.84

Table S2.

Uniprot ID	Protein Name	Gene name	BioID		
			Total controls	Total FKBP4	SAINT
P14618	KPYM	PKM2	0	1106	1.00
O43175	SERA	PHGDH	0	482	1.00
P55209	NP1L1	NAP1L1	6	377	1.00
P07737	PROF1	PFN1	0	341	1.00
Q86VP6	CAND1	CAND1	0	296	1.00
P12004	PCNA	PCNA	0	254	1.00
Q9P2J5	SYLC	LARS	0	244	1.00
Q15185	TEBP	PTGES3	0	212	1.00
Q01813	PFKAP	PFKP	9	207	1.00
P00558	PGK1	PGK1	0	201	1.00
P78417	GSTO1	GSTO1	0	173	1.00
O43852	CALU	CALU	0	171	1.00
Q96QK1	VPS35	VPS35	0	171	1.00
Q15785	TOM34	TOMM34	0	155	1.00
P42704	LPPRC	LRPPRC	0	154	1.00
P49915	GUAA	GMPS	0	139	0.97
P25398	RS12	RPS12	0	124	1.00
P30050	RL12	RPL12	20	109	0.99
Q9Y2Z0	SUGT1	SUGT1	0	92	1.00
Q9Y678	COPG1	COPG1	0	84	0.91
O43776	SYNC	NARS	0	83	0.89
Q9UNE7	CHIP	STUB1	3	82	1.00
P60891	PRPS1	PRPS1	0	82	1.00
P48444	COPD	ARCN1	0	71	0.99
P24666	PPAC	ACP1	0	71	1.00
P30566	PUR8	ADSL	0	70	1.00
Q9BTT0	AN32E	ANP32E	0	70	1.00
Q8N1F7	NUP93	NUP93	0	66	1.00
P14324	FPPS	FDPS	0	63	1.00
P61081	UBC12	UBE2M	0	62	0.97
Q96P70	IPO9	IPO9	3	59	1.00
P19367	HXK1	HK1	0	57	1.00
Q15293	RCN1	RCN1	0	54	1.00
O75131	CPNE3	CPNE3	0	53	0.99
Q99615	DNJC7	DNAJC7	0	52	1.00
O00443	P3C2A	PIK3C2A	0	52	1.00
Q9BRA2	TXD17	TXNDC17	0	49	1.00
Q92530	PSMF1	PSMF1	0	42	1.00
P46782	RS5	RPS5	0	40	1.00
P08559	ODPA	PDHA1	0	34	0.96
P13489	RINI	RNH1	0	29	1.00
O75436	VP26A	VPS26A	0	29	1.00
P17858	PFKAL	PFKL	0	29	0.97
O60684	IMA7	KPNA6	0	28	1.00

P25789	PSA4	PSMA4	0	28	1.00
Q9UBE0	SAE1	SAE1	0	26	1.00
Q9Y696	CLIC4	CLIC4	0	25	1.00
Q8TEX9	IPO4	IPO4	0	25	1.00
O15067	PUR4	PFAS	0	25	0.86
Q93008	USP9X	USP9X	0	22	1.00
P61586	RHOA	RHOA	0	21	0.87
P51570	GALK1	GALK1	0	19	1.00
P52788	SPSY	SMS	0	19	1.00
P09543	CN37	CNP	0	17	0.85
P43378	PTN9	PTPN9	0	16	1.00
Q6NXE6	ARMC6	ARMC6	0	16	1.00
Q9UEW8	STK39	STK39	0	13	0.99
Q9UBQ7	GRHPR	GRHPR	0	12	1.00
Q9BSH4	TACO1	TACO1	0	12	0.99
Q9UMR2	DD19B	DDX19B	0	10	0.99
Q96L21	RL10L	RPL10L	0	10	0.97
Q9GZT3	SLIRP	SLIRP	0	9	0.95
Q6UB35	C1TM	MTHFD1L	0	8	0.95
P50213	IDH3A	IDH3A	0	7	0.86
